# Hearing impairment and audiovisual speech integration ability: a case study report

**DOI:** 10.3389/fpsyg.2014.00678

**Published:** 2014-07-01

**Authors:** Nicholas Altieri, Daniel Hudock

**Affiliations:** Department of Communication Sciences and Disorders, Idaho State UniversityPocatello, ID, USA

**Keywords:** audiovisual speech integration, hearing impairment, capacity, processing speed, speech reading, lip-reading

## Abstract

Research in audiovisual speech perception has demonstrated that sensory factors such as auditory and visual acuity are associated with a listener's ability to extract and combine auditory and visual speech cues. This case study report examined audiovisual integration using a newly developed measure of *capacity* in a sample of hearing-impaired listeners. Capacity assessments are unique because they examine the contribution of reaction-time (RT) as well as accuracy to determine the extent to which a listener efficiently combines auditory and visual speech cues relative to independent race model predictions. Multisensory speech integration ability was examined in two experiments: an open-set sentence recognition and a closed set speeded-word recognition study that measured capacity. Most germane to our approach, capacity illustrated speed-accuracy tradeoffs that may be predicted by audiometric configuration. Results revealed that some listeners benefit from increased accuracy, but fail to benefit in terms of speed on audiovisual relative to unisensory trials. Conversely, other listeners may not benefit in the accuracy domain but instead show an audiovisual processing time benefit.

## Introduction

While a listener's hearing ability certainly influences language performance, decades of research has revealed that cues obtained from the visual modality affect speech recognition capabilities (e.g., Sumby and Pollack, 1954; McGurk and MacDonald, 1976; Massaro, 2004). One common example is the classic *McGurk effect* in which incongruent or mismatched cues from the visual modality (e.g., auditory /ba/plus visually articulated “ga”) influence auditory perception. Similarly, being able to see a talker's face under degraded listening conditions has been shown to facilitate both accuracy (Sumby and Pollack, 1954) and speed (e.g., Altieri and Townsend, 2011) compared to auditory-only recognition.

Auditory perceptual abilities are also associated with performance in the visual modality, as well as multisensory integration skills (Grant et al., 1998; Erber, 2003).

### Hearing loss and multisensory speech cues

#### High frequency hearing-loss

**Figure 1:**
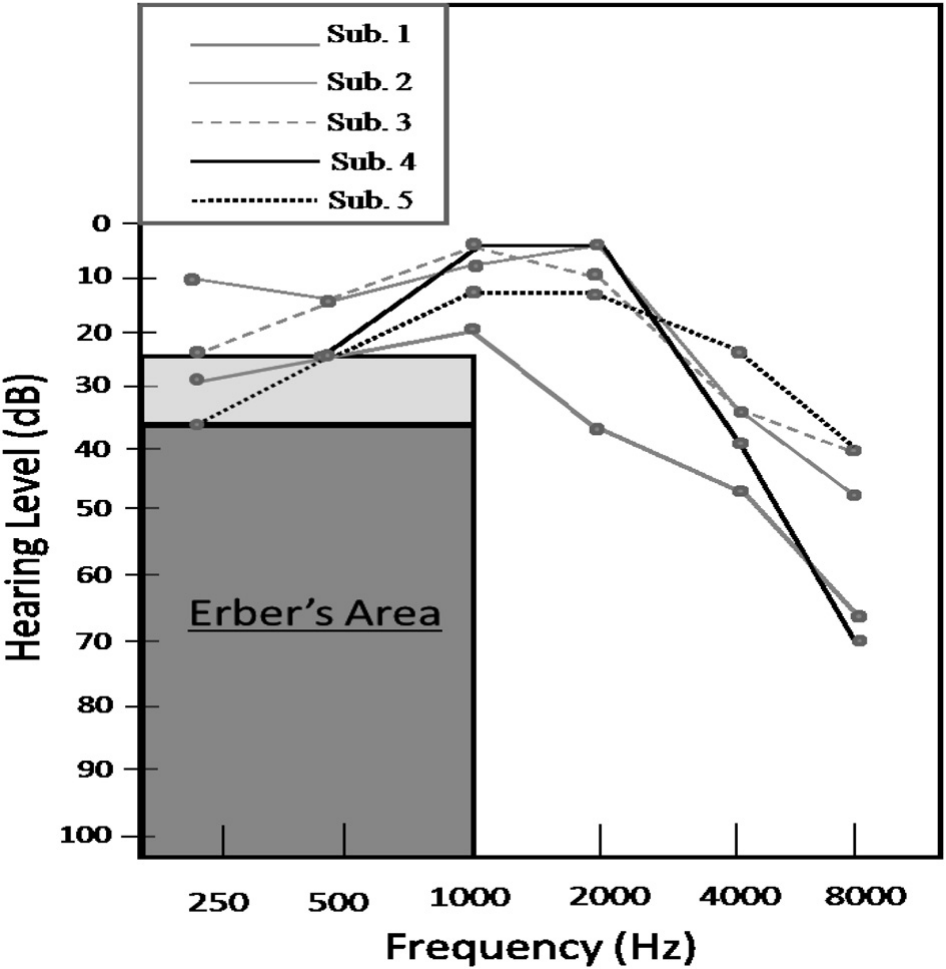
**Audiograms obtained from the five listeners with hearing loss**. The gray area represents Erber's area, while the light gray area immediately above indicates a range of mild to moderate low frequency hearing impairment.

Research has consistently indicated that face-to-face communication capabilities are impacted less by hearing loss compared to auditory-only perception. The frequency range of hearing loss also influences audiovisual integration and social conversational ability. While high-frequency hearing loss at frequencies greater than 1000 Hz has an adverse effect on auditory-only perceptual abilities, audiovisual perceptual skills appear to be less adversely affected, as noted by Erber (2002), (2003) among others (e.g., Danhauer et al., 1985). This observation is noteworthy considering that a significant proportion of older adults experience a progressive high-frequency hearing loss commonly known as *presbycusis*. Prototypical audiograms indicative of presbycusis remain generally flat in the frequency range up until approximately 1000 Hz and slope progressively downward at higher frequencies. (As depicted in Figure 1, we shall refer to an audiogram showing evidence for only high-frequency hearing loss as a “sloping” audiogram). Importantly, people with high-frequency hearing loss have been reported to generally retain the ability to obtain low-frequency cues from the auditory signal including: manner of articulation, voicing, nasality, and vowel information (e.g., Erber, 2003). However, the perception of high-frequency speech sounds, such as fricatives (e.g., /∫/), is affected to varying degrees.

For adults with hearing loss, being able to see a talker's face can therefore prove exceedingly helpful in terms of enhancing accuracy. Facial movements—especially those associated with high-frequency sounds such as place of articulation—can “fill in” for auditory speech cues that have become degraded (Erber, 1975). As an example, distinguishing between a bilabial vs. an alveolar stop (“ba” vs. “da”) is often straightforward in the visual modality, but often proves difficult auditorially. This becomes most noticeable when the quality of the auditory input is poor due to a noisy listening environment, hearing loss, or a combination of these factors.

#### Low-frequency hearing loss

Relationships between low-frequency hearing acuity and speech recognition have also been reported. Erber (2002), (2003) reported that “normal-hearing listeners”—those with low-frequency hearing thresholds better than 20 dB HL—have little difficulty hearing spoken words at a normal conversational distance and a volume level of 70 dB HL. Listeners with hearing loss greater than or equal to approximately 25 dB HL often fail to recognize auditory linguistic cues about manner of articulation and vowels to varying degrees, while listeners with thresholds higher than 50 dB HL usually fail to accurately perceive any speech sounds through the auditory modality. Research has hence indicated that listeners with “flat audiograms,” evidencing both low and high-frequency hearing loss may be poor integrators of audiovisual speech signals. This is ostensibly due to the fact that such listeners have a significantly reduced ability to isolate cues from the auditory signal related to voicing, nasality, vowel quality, and low frequency information. Listeners with low-frequency thresholds falling between 20 and 50 dB HL will predictably show significant variability in their ability to not only extract cues from the auditory speech signal, but combine them with complementary or redundant visemes. Taken together, the degree of hearing loss and pattern of errors in the unisensory modalities should contribute to a listener's ability to integrate multisensory cues information (e.g., Grant et al., 1998).

### Assessing audiovisual speech integration

We will implement the capacity assessment measure (Altieri et al., 2014b), known as *C_I(t)*, that compares both accuracy and reaction times (RTs) to parallel independent race model predictions derived from auditory and visual-only speech recognition trials. We therefore argue that a complete procedure for assessing integration ability should include multiple relevant dependent measures, namely accuracy and RTs. Comparisons between obtained data and predictions derived from statistical models that serve as a null-hypothesis will also be included (*Independent Race Models*; Miller, 1982). A RT-only measure of capacity, *C(t)*, (see Townsend and Nozawa, 1995) will also be assessed for each listener to separately diagnose RT capabilities. These methodologies are described further in Supplementary Material and by Altieri et al. (2014b). Generally, when capacity violates independent race model predictions at any time point (capacity < ½ or capacity > 1; Townsend and Wenger, 2004; Eidels et al., 2011; Otto and Mamassian, 2012) it indicates dependencies between auditory and visual modalities, and the presence of “integration.” When independent predictions are not violated, it still may indicate that listeners benefit from visual information in a statistical manner simply due to the availability of a greater number of cues (i.e., AV vs. A or V-only). For instance, if a listener “misses” auditory phonemic cues, they still have the opportunity to obtain a relevant cue from the visual modality even if the visual modality does not facilitate auditory processing *per se*.

The predictive power of capacity is becoming increasingly established. First, research has shown that capacity is a superior predictor of cognitive performance, neural functioning, and recognition capabilities compared to mean accuracy and mean RTs (Wenger et al., 2010). Additionally, even RT-only capacity measures have been demonstrated to be predictive of multisensory gain in terms of accuracy (Altieri and Townsend, 2011), as well as EEG measures of audiovisual integration (Altieri and Wenger, 2013) and multisensory learning (Altieri et al., 2014a). Finally, research using a sample of listeners without reported hearing loss showed that *C(t)* and *C_I(t)* scores were associated with l pure tone thresholds as well as traditional accuracy-based assessments of audiovisual gain (Altieri and Hudock, 2014).

This study will illustrate how speed-accuracy tradeoffs occur by implementing a novel procedure for measuring a listener's integration skills that was recently applied to a group of normal-hearing listeners, and those with only mild hearing loss (Altieri and Hudock, 2014). This study here will go a step further by assessing integration efficiency (i.e., capacity) in hearing impaired listeners. The five case studies consist of listeners with self-reported hearing loss of different ages, and with varying degrees of high and low-frequency hearing-loss as measured by auditory pure-tone thresholds. In the following experiments, differences in audiovisual processing speed or accuracy compared to unisensory performance will be the primary means used to measure capacity qua integration efficiency. Our aim was to illustrate how listeners with high or low-frequency hearing loss can systematically differ from each other in their ability to benefit from combined audiovisual cues in the accuracy and processing time domains. In doing so, we sought to identify how tradeoffs in speed and accuracy occur in listeners with prototypical audiograms evidencing high or low-frequency hearing loss.

#### Hypotheses

Listeners with auditory sensory deficits should adopt certain predictable strategies when processing audiovisual speech stimuli to maximize multisensory benefit. In terms of speed-accuracy strategies, some individuals may be slower on audiovisual trials relative to independent race model predictions in order to take advantage of visual speech cues. However, they may also be substantially more accurate and potentially show evidence for super-capacity and efficient integration (i.e., in Experiment 2). Additionally, we predict that these listeners should show greater audiovisual gain in open-set sentence recognition tasks (i.e., in Experiment 1). This scenario should often emerge in listeners with mild low-frequency and moderate high-frequency hearing difficulty. This type of hearing loss leads to mild degradation of certain vowel cues, and high-frequency information related to place of articulation. In these cases, auditory accuracy should be bolstered by visual speech cues. In fact, Altieri and Hudock (2014) showed that *C_I(t)* was correlated with low-frequency thresholds.

Second, we predict a larger RT-capacity gain (*C(t)*) for listeners with mild to moderate high-frequency hearing loss, but normal low-frequency thresholds. This is because auditory-only processing in these listeners should be slower but not significantly less accurate compared to normal-hearing listeners (due to the availability of more low-frequency vowel cues). This will, however, allow for complementary visual cues to facilitate speech recognition in the processing time domain especially. Generally, there should be a range in which auditory-only recognition becomes difficult, but enough cues are present in the signal to permit the combination of redundant and complementary visual cues to facilitate accuracy, perhaps in addition to speed. In future research, we predict that subjects with hearing loss will generally show superior integration skills compared to normal-hearing listeners, either in the speed, accuracy domain, or both.

## Methods

### Participants

This study analyzed data obtained from five listeners recruited from the Idaho State University campus in Pocatello, ID that demonstrated hearing impairment on their audiogram which was obtained prior to the study (average pure tone threshold ≥25 dB SPL). The measured hearing loss could either occur for low-frequencies, high-frequencies, or both. Each of the five participants was a native speaker of an American English dialect. Each participant reported normal or corrected 20/20 vision^1^. The same listeners participated in both Experiments 1 and 2. This study was approved by the Idaho State University Human Subjects Committee, and each participant was paid 10 dollars per hour for their participation. This study required approximately 60 min to complete. Participant information, such as average low and high-frequency hearing thresholds, gender, and age is shown in Table 1.

**Table 1:** **Information for each of the five listeners, including average low and high-frequency pure tone threshold**.

**Participant**	**Age**	**Gender**	**Low frequency**	**High frequency**	**Hearing loss**	**Hearing aid**
1	63	M	25	50	Sensory neural	Yes
2	22	F	8	35	Conductive	No
3	24	M	15	32	Sensory neural	No
4	60	M	17	37	Sensory neural	No
5	72	F	25	25	Sensory neural	No

### Audiometric testing

Pure-tone hearing thresholds were obtained for each volunteer prior to participation in this study using an *Ambco 1000 Audiometer*. Hearing thresholds were obtained in a sound attenuated chamber. Thresholds were obtained for 250, 500, 1000 (low frequencies), and, 2000, 4000, and 8000 Hz (high-frequencies; Erber, 2003) tones separately to each ear using headphones. For each frequency, thresholds were obtained via the presentation of a continuous tone. The following standard staircase procedure was used: when the listener identified the tone correctly by button press, the sound level was reduced 10 dB. If they failed to correctly indicate the presence of the tone, the decibel level was raised by 5 dB on the subsequent presentation.

### Experiment 1: open-set sentence recognition

#### Stimuli

The sentence stimuli used in Experiment 1 consisted of 75 sentences obtained from a database of recorded audio-visual sentences from the CUNY database (Boothroyd et al., 1985). Each of the sentences was spoken by a female talker. The stimulus set included 25 audiovisual, 25 auditory, and 25 visual-only sentences. The stimuli were obtained from a laser video disk and rendered into a 720 × 480 pixel video, digitized at a rate of 30 frames per second. Each stimulus was displayed on a standard Dell computer monitor with a refresh rate of 75 Hz. The auditory track was removed from each of the sentences using Adobe Audition for the visual-only sentences, and the visual component was removed for the auditory-only block. The sentences were subdivided into the following word lengths: 3, 5, 7, 9, and 11 words with five sentences for each length for each stimulus set (Altieri et al., 2011). This was done because sentence length naturally varies in conversational speech. Sentences were presented randomly for each participant, and we did not provide cues regarding to sentence length or semantic content. The sentence materials are displayed in Supplementary Material. To avoid ceiling performance, the auditory component of the signal was degraded using an 8-channel sinewave cochlear implant simulator (AngelSim: http://www.tigerspeech.com/). Consistent with Bent et al. (2009), we selected the following settings for the CI simulator: band pass filters were selected to divide the signal into eight channels between 200 and 7000 Hz (24 dB/octave slope), and a low pass filter was used to derive the amplitude envelope from each channel (400 Hz, 24 dB/Octave slope). Cochlear implant simulation with this number of channels generally leads to accuracy scores of approximately 70% words correct in young normal-hearing listeners in sentence recognition. Furthermore, it yields similar accuracy as multi-talker babble background noise (Bent et al., 2009).

#### Procedure

Accuracy data from the 75 audiovisual (25), auditory-only (25), and visual-only (25) sentences listen in Supplementary Material were obtained from each participant. Trials were presented in separate blocks consisting of 25 audiovisual, 25 auditory, and 25 visual-only trials. The order of audiovisual, auditory, and visual-only block presentation was randomized across participants in an effort to avoid order effects. The stimuli in both experiments were presented to the participants using E-Prime 2.0 (http://www.pstnet.com/eprime.cfm) software.

Participants were seated in a chair approximately 24 inches from the monitor. Each trial began with the presentation of a black dot on a gray background, which cued the participant to press the space bar to begin the trial. Stimulus presentation began with the female talker speaking one of the sentences. After the talker finished speaking the sentence, a dialog box appeared in the center of the monitor instructing the participant to type in the words they thought the talker said by using a keyboard. Each sentence was given to the participant only once, and feedback was not provided on any of the trials. Scoring was carried out in a manner similar to the protocol described by Altieri et al. (2011). Whenever the participant correctly typed a word, then that word was scored “correct.” The proportion of words correct was scored in each sentence. Word order was not a criterion for a word to be scored correctly, and typed responses were manually corrected for misspellings. (Upon inspection of the data, participants did not switch word order in their typed responses.). As an example, for the sentence “Is your sister in school,” if the participant typed “Is the…” only the word “Is” would be scored as correct, making the total proportion correct equal to 1/5 or 0.20.

### Experiment 2: speeded word recognition: capacity analysis

#### Stimuli

The stimulus materials consisted of audiovisual movie clips consisting of two female talkers. The stimuli were obtained from the Hoosier Multi-Talker Database (Sherffert et al., 1997). Two recordings of each of the following monosyllabic words were obtained from two female talkers: *Mouse, Job, Tile, Gain, Shop, Boat, Page*, and *Date*. These stimuli were drawn from similar studies carried out by Altieri and Townsend (2011), and also by Altieri and Wenger (2013). The auditory, visual, and audiovisual movies were edited using *Adobe After Effects*. Each of the auditory files was sampled at a rate of 48 kHz (16 bits). Each movie was digitized and rendered into a 720 × 480 pixel clip at a rate of 30 frames per second. Similar to Experiment 1, the auditory component signal was degraded using the 8-channel sinewave cochlear implant simulator. The duration of the auditory, visual, and audiovisual files ranged from 800 to 1000 ms. A previous report demonstrated that the variation in the duration of the movies did not influence RTs; rather, linguistic factors such as the confusability of the auditory and visual phonetic cues proved to be a major factor affecting processing speed (Altieri and Wenger, 2013). For example, the words “job” and “shop” were difficult to distinguish visually, and hence, visual-only RTs for these stimuli were slower and less accurate compared to the other words. Conversely, “boat” and “gain” were significantly easier to distinguish due to the difference in place of articulation.

#### Procedure

The audiovisual, auditory, and visual only trials were presented randomly in one block. There were a total of 128 audiovisual trials (64 spoken by each talker, where each of the 8 words was repeated 8 times per talker), 128 auditory-only trials, and 128 visual-only trials, for a total of 384 experimental trials. This portion of the experiment required 20–30 min to complete. Experimental trials began with a white dot on a gray background appearing in the center of the monitor. Each trial consisted of auditory-only, visual-only, or audiovisual stimuli. Auditory stimuli were played at a comfortable listening volume (approximately 70 dB SPL) over *Beyer Dynamic-100* Headphones.

Responses were collected via button press using a keyboard. Each of the buttons, 1–8, was arranged linearly on the keyboard and was labeled with a word from the stimulus set. Participants were instructed to press the button corresponding to the word that they judged the talker to have said as quickly and accurately as possible. Responses were timed from the onset of the stimulus on each trial. Inter-trial intervals randomly varied on a uniform distribution between 750 and 1000 ms. On auditory-only trials, participants were required to base their response solely on auditory information, and on visual-only trials participants were required to lip-read to make the speeded response. Auditory-only trials were played with a blank computer screen. Similarly, visual-only trials were played without any sound coming from the speakers. Each listener received 48 practice trials at the onset of each experimental block to assist with learning the response mappings on the keyboard.

## Results and discussion

### General summary: RT and accuracy

The results from the open-set sentence recognition experiment (Experiment 1) are shown in Table 2 and the results for the speeded-word recognition task (Experiment 2) are shown in Table 3. The tables show auditory- (A) and visual- (V) only percent correct for the CUNY sentences, respectively, as well as the predicted $\hat{p}$(*AV*) = *p*(*A*) + *p*(*V*) − *p*(*A*) * *p*(*V*) and obtained audiovisual (AV) scores. The actual AV Gain scores are also displayed (AV_Gain_ = *p(AV)* − *max*{*p(A)*, *p(V)*}; cf. Altieri and Wenger, 2013). For comparison purposes, the mean auditory, visual-only and audiovisual accuracy scores and the predicted and obtained AV integration scores are displayed for normal-hearing participants selected from another study (Altieri and Hudock, 2014).

**Table 2:** **CUNY sentence recognition scores for each listener**.

**Listener**	**A**	**V**	**Predicted AV**	**Obtained AV**	**AV gain**
Average	78.40(7.20)	14.50(7.80)	81.40(6.80)	95.00(3.10)	16.60(6.80)
1	57	13	63	95	38
2	51	14	58	93	42
3	63	0	63	91	28
4	59	13	64	88	29
5	71	11	74	94	23

**Table 3:** **Speeded word recognition accuracy scores, mean RTs, and standard deviations (parentheses)**.

**Listener**	**A**	**V**	**Predicted AV**	**AV**	**Max(*C_I(t)*)**	**Max(*C(t)*)**	**AV_RT**	**A_RT**	**V_RT**
Average	98	73	99	98	1.28	1.40	1812 (284)	1823 (291)	2432 (514)
1^*^	55	30	69	95	5.40	0.49	1602 (244)	1993 (601)	1509 (353)
2	97	77	98	99	1.34	1.19	1790 (529)	1775 (549)	2124 (489)
3	99	80	99	100	0.84	0.51	2091 (521)	1875 (473)	3297 (1301)
4	100	70	100	99	1.41	2.47	2258 (899)	2332 (627)	3312 (1621)
5	98	51	99	99	1.10	1.20	2126 (545)	2129 (542)	2877 (811)

The standard deviation (SD) for the normal-hearing listeners was calculated across individual listeners. The

“* indicates lower auditory and visual-only accuracy for this listener, along with considerably higher C_I(t).

Table 2 reveals that the two listeners with the lowest auditory-only accuracy (i.e., 1 and 2) showed the highest audiovisual gain, as predicted. Both listeners yielded gains that were greater than 2.5 SDs from the control participants. Interestingly, Table 3 showed very low auditory-only accuracy for Participant 1. This listener reported difficulty in distinguishing several words that differed on key high-and low-frequency characteristics such as certain consonants (e.g., “job” vs. “shop”), and vowels, respectively (e.g., “mouse” vs. “boat”). Another feature of this listener was the comparatively fast visual-only responses. It appears that this listener responded “fast” on visual-only trials in order to get the trial finished quickly, perhaps because he was aware of the difficulty in accurately identifying content. However, when visual cues were combined with auditory, the listener was able to slow down in order to effectively merge the information with auditory cues. These findings will be further explored in the subsequent capacity analysis.

**Figure 2:**
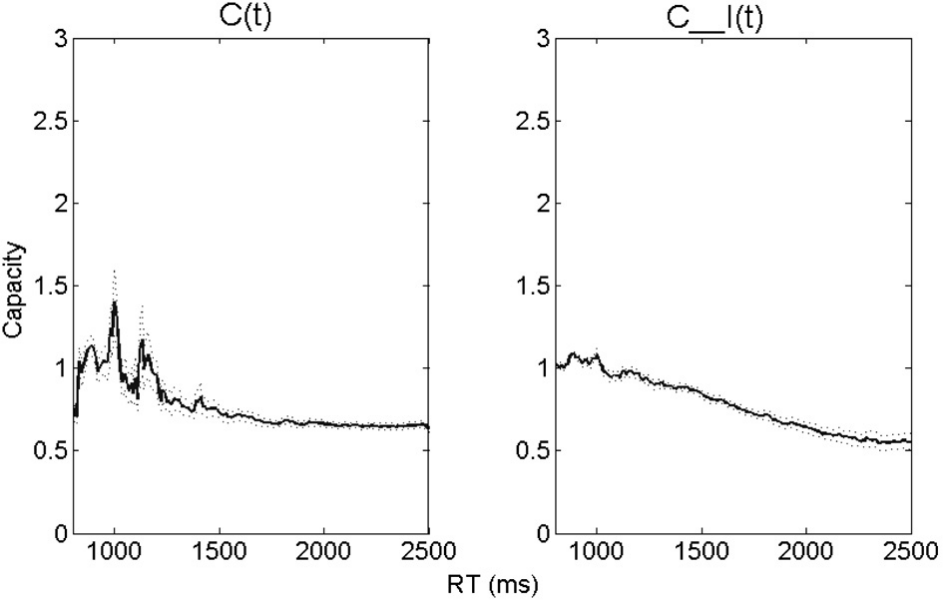
**A similar figure is displayed in Altieri and Hudock (2014), after some listeners with mild hearing impairment (thresholds > 25 dB) were removed (*n* = 39)**. It shows mean capacity-integration efficiency measures (thick solid line = Capacity/Integration Efficiency). The left panel shows *C(t)*, and the right panel shows *C_I(t)*. The dotted lines show one standard error (SE) of the mean.

Next, the average capacity data for a group of normal-hearing participants are displayed in Figure 2. Together, these values denote the capacity-integration measures obtained at each time point. The RT-only *C(t)* values are displayed in the left panel, and the RT-accuracy *C_I(t)* values on the right. The dotted lines indicate one standard error (SE) of the mean.

**Figure 3:**
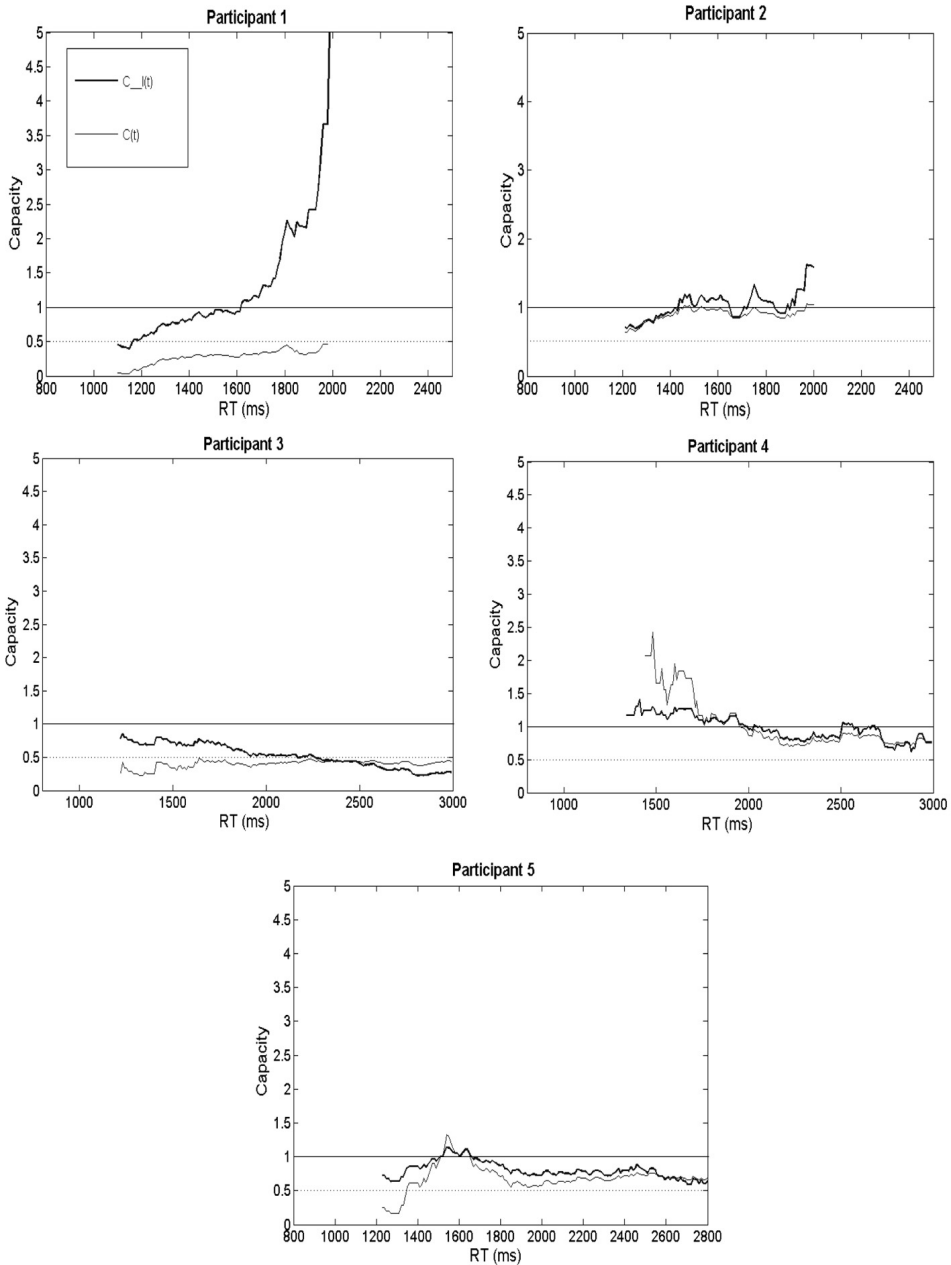
**Capacity (*C(t)* and *C_I(t)*) plotted individually for each of the five listeners**.

Figure 3 shows capacity separately for hearing-impaired listeners 1 through 5. In each plot, the thick line shows *C_I(t)*, while the lighter line shows the RT-only *C(t)*. The actual capacity value at a time point is shown on the y-axis and the RT is displayed on the x-axis. To facilitate discrimination between strong integration ability (super-capacity) vs. poorer integration ability (limited capacity), two separate bounds can be found in each panel. First, limited capacity is defined by the dotted line at capacity = ½; the reason is that if processing resources were evenly divided between channels, then we would predict that the energy expended on AV trials would be half of the sum expected from A plus V efficiency (Townsend and Nozawa, 1995). Such a scenario would result if multisensory interactions between auditory and visual modalities were present, but the visual signal inhibited auditory recognition (e.g., Eidels et al., 2011). A useful heuristic bound separating unlimited and super-capacity corresponds to capacity = 1; the reason is that the race model inequality predicts capacity equal to 1 if processing on AV trials equals the sum of A and V processing (Townsend and Nozawa, 1995).

Crucially, the difference between *C_I(t)* and *C(t)* at any specific point, along with accuracy, provides information about a participant's processing strategy and integration ability (Altieri et al., 2014b). This is because *C(t)* furnishes information about speed (is the listener able to take advantage of visual speech information in terms of speed?). Also, obtained AV accuracy measured in comparison to the formula, $\hat{p}$(*AV*) = *p(A)* + *p(V)* − *p(A)* * *p(V)* furnish information about whether integration is efficient or inefficient in the accuracy domain. Suppose *C_I(t)* is greater than 1 indicating efficient integration. Further, suppose that *C(t)* is also greater than 1 and accuracy equals independent race model predictions. This scenario would indicate that the listener is an efficient integrator due to the ability to take advantage of processing time on audiovisual trials rather than accuracy *per se*. Analogous logic applies to other scenarios in which the listener may show evidence for *C_I(t)* > 1, but slows down to obtain higher than predicted audiovisual accuracy (*C(t)* < 1). We shall now discuss capacity results for the individual hearing-impaired listeners by examining *C_I(t)*, *C(t)*, and the discrepancy between predicted and obtained audiovisual accuracy shown in Table 3.

#### Case 1

This first case involves a 63 year-old male with mild-moderate sudden-onset bilateral high and low-frequency hearing loss of unknown origin. The average pure tone threshold for the low frequency tones (250–1000 Hz) was approximately 20–25 dB HL, while the average threshold for the high frequency tones (2000–8000 Hz) was 45–50 dB HL. This listener wears bilateral hearing aids to facilitate everyday perception, but did not use a hearing aid during testing.

The upper left hand panel of Figure 3 (labeled “Participant 1”) shows both capacity measures (accuracy was reported in Table 3). Remarkably, *C(t)* was extremely limited and less than ½ for the preponderance of time points. This finding is intriguing because it also shows that for each point in time, that Participant 1's *C(t)* was less than the average obtained from the group of normal-hearing volunteers (left panel of Figure 2). It indicates that Participant 1 slowed down on audiovisual trials compared to independent race model predictions. Integration measured in terms of speed (*C(t)*) was thus exceptionally poor for this listener. This change in speed appears to have resulted from a speed-accuracy tradeoff on visual-only trials since the listener was faster than average, but showed poor accuracy. However, when visual information was combined with the auditory modality, the listener proved capable of taking advantage of the combined auditory-visual information by slowing down to achieve better audiovisual accuracy. When both speed and accuracy were taken into account, Participant 1 displayed evidence for superior integration abilities across most times—particularly slower RTs. This listener elicited greater integration efficiency than normal-hearing participants, and for times greater than 1600 ms post stimulus onset, also demonstrated super-capacity. As observed from the data in Table 3, this higher than predicted audiovisual accuracy (Predicted = 0.69; Obtained = 0.95) rather than speed drove integration ability.

While this listener had mild low-frequency hearing loss and thus generally poor performance in the auditory domain, he appeared to compensate by slowing down on audiovisual trials relative to visual-only trials in order to maximize accuracy—thus optimizing integration and residual perceptual matching strategies. Significantly, capacity was also reflected by the substantial audiovisual gain on sentence recognition. While auditory-only CUNY sentence recognition was poor due to hearing loss, gain scores were substantially high and only exceeded by Participant 2. This listener's integration skills predict strong face to face communication ability, resulting from the ability to obtain relevant cues from the auditory modality and combine them with visual information. Nonetheless, hearing aids likely facilitate speech communication by providing additional auditory cues, which should help speed-up information processing in difficult listening environments.

#### Case 2

Case number 2 involved a 22 year old female with bilateral high-frequency bone conductive hearing loss resulting from surgery as a toddler. This listener reported that she never used a hearing aid. The average low frequency threshold was 8 dB, while the average high-frequency threshold was approximately 35 dB HL.

The panel in Figure 3 labeled “Participant 2” displays the capacity results. The RT only *C(t)* data show evidence for unlimited capacity across most time points, with the exception of very fast processing times. This indicates that capacity was consistent with independent race model predictions since *C(t)* was approximately unlimited. This finding indicated that Participant 2 was capable of statistically benefiting from visual cues in the RT domain. Furthermore, *C(t)* was higher compared to the average obtained from the normal-hearing listeners, and also higher than Participant 1's.

One explanation for this particular finding was that the degraded consonant information contributed to slower auditory-only responses and poor auditory-only sentence recognition capabilities; however, enough low-frequency vowel information was present for the listener to enable correct unisensory recognition in the context of a small set size, albeit at a slower pace. When both auditory and visual cues were present, this listener adequately matched the visual to the auditory cues to compensate for the deficits in auditory recognition, and hence, achieved borderline efficient integration. This processing speed difference was perhaps mirrored by the substantial accuracy gain achieved in open-set sentence recognition (Table 2). When combining the relative efficient integration in the time domain with accuracy levels in the forced choice task (Predicted = 0.98; Obtained = 0.99), overall integration skills indexed by *C_I(t)* shows evidence for unlimited capacity that was driven by RTs. Similar to *C(t), C_I(t)* was greater than the average obtained from the normal-hearing listeners.

#### Case 3

Case number 3 was a 24 year old male with mild to moderate high-frequency sensory neural hearing loss—reportedly due to repeated noise exposure while serving in the military. This listener did not use a hearing aid. His audiogram showed that the average low frequency threshold in the better ear was 15 dB HL, while the average high-frequency threshold was approximately 30 dB HL.

Unlike the participant in case 2 who is of similar age and hearing ability, an overview of Participant 3's capacity measures revealed poor integration skills—in part due to poor visual-only skills as reflected in the CUNY sentence recognition task (see Table 2). Furthermore, this listener was slow but accurate on visual-only trials, and slower on audiovisual compared to auditory-only trials in the speeded word recognition task. These results perhaps indicate the presence of inhibition from the visual modality (Altieri and Townsend, 2011; Altieri and Wenger, 2013). Hence, for two listeners with similar demographics and thresholds, one observes differences in capacity that may be due to differences in hearing history, or other perceptual capabilities (e.g., visual-only). As one may observe in the panel labeled “Participant 3,” both capacity measures are lower than the normal-hearing average, as well as Participant 2's for many time points. The *C(t)* hovers around the lower bound for fixed capacity indicating that this listener is “slow” when extracting visual place of articulation cues and filling in for degraded consonant information in the auditory modality. The results revealed similar auditory-only mean RTs for Participants 2 and 3, although significantly slower audiovisual RTs for Participant 3.

Crucially, the unitary *C_I(t)* measure was slightly higher than *C(t)*, although it was lower than 1, and lower than the bound on fixed capacity for his slowest RTs. The reason that the overall integration metric of *C_I(t)* showed evidence for slightly better integration compared to *C(t)* was that obtained accuracy levels approximated predicted scores in the speeded word task, making up for sluggish audiovisual RTs. Additionally, this participant showed substantial audiovisual gain on CUNY sentences, indicating the ability to benefit from visual information when combined with auditory speech cues under certain circumstances. Taken together, it appears that this participant has the ability to benefit from multisensory cues although he may fail to benefit in the processing time domain.

#### Case 4

Case number 4 included a 60 year old male with moderate age related high-frequency hearing loss. This listener was aware of his hearing loss, but did not use a hearing aid to facilitate language perception. His average low frequency threshold in the better ear (right) was 17 dB, while the average high-frequency threshold was recorded as 37 dB.

Similar to the listener from case 2, this listener revealed a strong correspondence between *C(t)* and *C_I(t)*. This participant showed evidence of unlimited capacity due to the fact that both capacity measures corresponded to independent model predictions for the vast majority of time points. Audiovisual responses appeared slightly faster than race model predictions for early recognition times. Audiovisual mean RTs were faster on average compared to auditory only RTs by approximately 100 ms, and also faster than visual-only RTs, which were quite sluggish. This listener's visual-only perception on both the CUNY sentence perception and speeded word recognition tasks were in the normal range at 13 and 70% correct, respectively; however, the visual-only RTs suggest that he slowed down to achieve this accuracy level. Interestingly, the observation of super-capacity, and that the audiovisual trials were processed faster than auditory-only trials, indicates that visual information about place of articulation sped-up auditory recognition.

Similar to listener 2, this listener exhibited an integration profile that was consistent with race model predictions. Overall, his integration was superior to the normal-hearing average, which should often be true of listeners with mild to moderate hearing-loss. Despite the relatively poor auditory-only performance due to the loss of high-frequency cues, this listener's integration and lip-reading skills should facilitate face-to-face conversation enough to reduce or eliminate the need for a hearing aid.

#### Case 5

Case number 5 was a 72 year old woman with a flat audiogram showing evidence for bilateral mild hearing loss. Her average low and high-frequency hearing thresholds in the better ear were measured at approximately 20–25 dB HL. This listener reported being unaware of her hearing loss, and consequently, did not use a hearing aid.

Unlike Participant 1's, the capacity results showed slightly limed to unlimited capacity or mildly inefficient integration for responses slower than 1500 ms, but close to unlimited capacity for faster responses. This observation places this listener at odds with the group of normal-hearing listeners who showed super-capacity for faster processing times when comparing this participant's results with those shown in Figure 2. The mildly inefficient integration appears to suggest that this listener failed to benefit in terms of speed from visual cues (the fastest audiovisual responses did not differ from the fastest auditory-only RTs, and were only slightly faster than the visual-only RTs). Although audiovisual RTs were generally sluggish compared to independent model predictions, accuracy was at ceiling, and equal to predictions.

Overall, the measured *C_I(t)* was only marginally less than 1 for the preponderance of recognition times since accuracy made up for the moderate deficits in speed. In fact, since this listener's integration ability approximates the skills of normal-hearing listeners for most time points, she will likely be an effective face-to-face communicator without the use of a hearing aid, except in challenging conversational environments.

## General discussion

The purpose of this study was to provide novel applications of a new capacity approach to identify loci of audiovisual speech integration abilities in hearing-impaired listeners. Specifically, this study extended recent capacity results using single point summaries (Altieri and Hudock, 2014) by illustrating how differences in auditory sensory acuity may be related to speech integration skills in listeners with different types of hearing impairment.

Of course, the picture appears somewhat complex as factors besides sensory acuity affect integration skills. Our results revealed how speech integration differs among hearing impaired individuals. Our results did demonstrate that older and younger listeners with different levels of hearing loss can yield similar capacity. For example, listeners 2 and 4 different in age (22 vs. 60 years old, respectively), and had different hearing loss etiology. In spite of these differences, integration skills as measured by accuracy and capacity were remarkably similar. On the other hand, listeners 1 and 5 were both over 60, and displayed similar audiograms; however, their integration skills differed both qualitatively and quantitatively. The upshot of these findings is that while relationships have been shown to emerge between sensory acuity and integration skills (e.g., Altieri and Hudock, 2014), pure tone thresholds are just one predictor of integration ability.

Cognitive factors also contribute to integration performance. In this report, however, we attempted to minimize the impact of higher cognitive factors at least, such as memory capabilities^2^. Yet, age may be one factor impacting integration abilities, as it has recently been shown to be correlated with capacity (Altieri and Hudock, 2014), and associated with poorer lip-reading (Sommers et al., 2005). One possibility is that many older listeners may effectively utilize the visual speech modality, but only in the context of sufficient auditory speech information. Therefore, we predict that older listeners with mild hearing loss may be efficient integrators—particularly in the accuracy domain. Audiovisual RTs, however, may be slower than independent model predictions in order to allow these listeners sufficient time to obtain cues and thus maximize accuracy. Interestingly, this speed-accuracy tradeoff associated with aging has been consistently reported by Ratcliff and colleagues across a variety of cognitive and perceptual tasks (e.g., Ratcliff et al., 2004). The results provided by Participant 1 yields key evidence for this hypothesis. Nonetheless, because we utilized case study methodology, conclusive statements about the relationship between variables such as hearing loss etiology, age, and audiometric configuration and integration skills are difficult to ascertain. Critically, such integration strategies would be impossible to uncover using most current approaches for assessing integration which rely on accuracy-only (Braida, 1991; Grant et al., 1998; Massaro, 2004).

### Assessing audiovisual speech integration

Our findings show promise inasmuch as they indicate the importance of incorporating comprehensive speed-accuracy assessment measures. Processing speed is one critical variable predictive of information processing abilities, and one that is well-known to be adversely affected by aging and of course hearing ability. Unfortunately, speed has been overlooked as a viable measure of integration (see Altieri and Townsend, 2011; Winneke and Phillips, 2011). Before the capacity approach can be incorporated into future audiological assessment protocols, key developments seem to be in order. Obtaining normative data on integration skills using *C_I(t)* and *C(t)* will be necessary. Another important development will involve collecting larger data sets consisting of hearing-impaired listeners with different audiometric configurations and different levels of cognitive functioning. Overall, capacity measures should prove important since audiologists do not comprehensively assess either visual or audiovisual processing capabilities in those suspected of hearing impairment, even though these skills are relevant for face-to-face communication capabilities.

## Summary and conclusion

This report provided a basis for a comprehensive methodological approach for examining speech integration in listeners with suspected hearing loss. Accuracy in open-set sentence recognition may be assessed subsequent to traditional audiometric testing. Next, the suggested approach would be to measure *C(t)* and *C_I(t)* using a closed-set speeded word recognition experiment to analyze the extent to which a listener benefits from multisensory cues relative to the predictions of independent models in which integration does not occur. Such a protocol will allow one to determine the locus of a listener's integration capabilities. For example, suppose a listener exhibits high *C_I(t)* in conjunction with low *C(t)*. This could indicate an inability to benefit from visual speech cues in terms of processing speed, but that slowing down may help one take advantage of visemes to achieve high accuracy.

Notwithstanding our findings, one may observe that while audiograms and auditory-only hearing ability may be associated with integration (Erber, 2002, 2003), individual differences in integration ability exist. Besides auditory sensory capabilities, other sensory or cognitive factors can influence integration ability. This includes age (Bergeson and Pisoni, 2004; Ratcliff et al., 2004; Sommers et al., 2005; Winneke and Phillips, 2011), complex visual-only perceptual abilities (i.e., face recognition), processing speed and related strategies, and working memory skills (Lunner et al., 2009). Interaction among these factors and how they relate to speech integration skills have only recently begun to be explored in a model-based way and therefore require considerable future investigation.

### Conflict of interest statement

The authors declare that the research was conducted in the absence of any commercial or financial relationships that could be construed as a potential conflict of interest.

## Footnotes

^1^Altieri and Townsend (2011) showed that substantial degradation of the visual speech signal (e.g., darkening by 90% using Final Cut Pro) is necessary to measurably affect recognition capabilities. This is because the visual cues used to detect the speech signal, such as visual place of articulation, are highly salient. Therefore, in listeners with normal or corrected vision, visual acuity should not have been a factor influencing audiovisual integration skills.

^2^All participants were employed or full time students and none of them reported any history of cognitive impairment or traumatic brain injury. Furthermore, each listener participated in a 5-min cued memory task involving a “study” and subsequent “test phase” (i.e., judging whether a picture was “old” or “new”). Each of the 5 participants scored 83% correct or better. The average score in a group of 84 participants (part of another study) was 91% correct with a standard deviation of 5.6%. Therefore, each listener was within 2.0 SDs of the mean of the larger sample of volunteers.
