# Supplementary material for: Hearing impairment and audiovisual speech integration ability: a case study report
Source: Front Psychol. 2014 Jul 1;5:678. doi: 10.3389/fpsyg.2014.00678 (PMC4076931; doi:10.3389/fpsyg.2014.00678)
Supplement: Supplementary file 1 [file DataSheet1.DOCX]

Appendix A

Assessment Methods for Audiovisual Integration

These measures were described by Altieri, Townsend, and Wenger (2014). They may thus be omitted at the discretion of the reader.

1. *Accuracy Methods*

As general practice, audiovisual integration has been assessed by comparing audiovisual accuracy (Bergeson & Pisoni, 2004; Erber, 1975; Grant, Walden & Seitz, 1998; Sumby & Pollack, 1954). One may compute audiovisual gain as follows: $AVGain= p\left( AV \right)- Max\left\{ p\left( A), p(V \right) \right\}$. The predicted AV accuracy, derived from race model predictions assuming independence between modalities is given by the following: $\hat{p}\left( AV \right)= p\left( A \right)+ p\left( V \right)- p\left( A \right)*p(V)$ (each ‘*p*’ denotes probability correct; *p(A)* indicates percentage correct on auditory-only trials). This latter method has more recently been described by Altieri, Townsend, and Wenger (2013), who also provided computer code for obtaining both capacity measures described next.

1. *Response Time Methods*

Integration has also been measured using response time (RT) distributions to compare audiovisual processing relative to *independent race model predictions* (Miller, 1982; Townsend & Nozawa, 1995); the intent is to provide a more in-depth assessment of recognition ability than can be provided by means (Altieri & Townsend, 2011; Winneke & Phillips, 2011). *Independent models* predict that auditory and visual sources are processed separately and independently from one another (Miller, 1982; Townsend & Nozawa, 1995). For statistical reasons, independent models predict that recognition times for audiovisual trials are at least as fast, or slightly faster than auditory-only or visual-only trials (for at least some processing intervals). To understand this type of statistical facilitation, suppose an audiovisual syllable such as “ba” is presented to a listener. However, further suppose that the signal is degraded and the listener cannot obtain enough auditory information on that trial to identify the syllable. However, when seeing the talker’s face, the listener sees the viseme “b” and correctly guesses “ba”. This makes the audiovisual trial comparatively faster than if only auditory information had been present. Hence, by virtue of the fact that two sources of information are available on these trials rather than just one, speed (and also accuracy, see *C_I(t)*) should be at least as good or slightly better than the best unisensory condition. This is what is referred to as “unlimited capacity” or “independent model predictions”.

This approach has been augmented by describing integration within the construct of *capacity* (Altieri & Townsend, 2011; Altieri et al., 2013). In the speech recognition literature, traditional methods for measuring integration have relied mostly on mean accuracy (Altieri, Pisoni, & Townsend, 2011; Bergeson & Pisoni, 2004); however, accuracy measures generally fail to capture the notion of efficiency and they lack information about processing rate.

To summarize, capacity has proven advantageous for clinical and research purposes because it has been shown to constitute a more sensitive measure of cognitive function, compared to simply computing mean accuracy or mean RTs, which lack the fine grained detail of an entire distribution (Wenger, Negash, Petersen, & Petersen, 2010). The information provided by distributions of RTs is advantageous for conceptual reasons (i.e., capacity ~ work completed), as well as statistical reasons since it is non-parametric, compares processing to independent model predictions, and also because integrated hazard functions contain more power than means (Townsend, 1990).

The method for calculating capacity involves first obtaining the empirical distribution of RTs from an experimental condition and computing the integrated hazard function (Townsend & Nozawa, 1995). As we shall see, this essentially involves obtaining a cumulative sum of RTs, and then computing the logarithmic transformation. Here, the cumulative hazard function is defined as: *H_AV_(t)* (*H(t)* = -log[1-*F(t)*], where *F(t)* corresponds to the empirical cumulative distribution function of RTs, and 1-*F(t)* denotes the probability that a response has not yet occurred. The numerator in the capacity equation below contains the integrated hazard function, *H(t)*, for each of the words presented audio-visually. The sum in the denominator denotes the “null-hypothesis independent model predictions” obtained from the unisensory A and V trials: .

This equation leaves us with three possible outcomes.

1) Capacity may be less than 1 as a result of slower multisensory processing compared to the responses from the unisensory conditions. This suggests limited capacity or inefficient integration since AV process is worse than would be predicted by independent race models (Altieri & Townsend, 2011; Townsend & Nozawa, 1995). 2) Capacity may be equal to 1, indicating that capacity equals null-hypothesis independent model predictions where A and V information do not interact. This corresponds to the predictions of independent race models and represents “borderline efficient” integration. 3) Capacity can be greater than 1 at a specific point in time, indicating faster responses in AV trials compared to A and V-only trials. This indicates super-capacity or efficient integration because AV processing is faster than independent model predictions.

*Combined Speed and Accuracy*

The focus in this case study research concerns a recent augmentation to the capacity measure, by allowing it to simultaneously incorporate both speed and accuracy when assessing efficiency relative to independent race model predictions (Townsend & Altieri, 2012; Altieri et al., 2013). Assessing both speed and accuracy in a unitary measure is beneficial because processing speed, and one’s ability utilize lip-reading information, are related to hearing ability and aging (Ratcliff, Thapar, & McKoon, 2004; Sommers, Tye-Murray, & Spehar, 2005; Tye-Murray, Sommers, & Spehar, 2007; Winneke & Phillips, 2011).

The logic for the new measure (referred to as *C_I(t)*) is as follows: suppose a listener is presented with an AV spoken word. The word is correctly identified as soon as the auditory, visual, OR both auditory and visual information are accurately recognized. Overall probability correct in the auditory or visual domains occurs via the following sum: Being correct on A at or before a certain time while being incorrect on V*,* plus being correct on V at or before a certain time while being incorrect on A, plus being correct on A at or before a certain time and correct on V (while recognition has not yet occurred yet on V), plus being correct on V at or before a certain time (while recognition has not yet occurred yet on A), plus the probability that correct recognition occurs in both A and V domains by a certain time:

$$\begin{matrix} \int_{0}^{t} P_{AV}(T_{AVC}=t'\cap t'<T_{AVI})dt'=\int_{0}^{t} P_{A}(T_{AC}=t'\cap t'<T_{AI})dt'*P_{V}(I)+\int_{0}^{t} P_{V}(T_{VC}=t'\cap t'<T_{VI})dt'*P_{A}(I) \\ +\int_{0}^{t} P_{A}(T_{AC}=t'\cap t'<T_{AI})dt'*\int_{t'=t}^{\infty} P_{V}(T_{VC}=t'\cap t'<T_{VI})dt'+\int_{0}^{t} P_{V}(T_{VC}=t'\cap t'<T_{VI})dt'*\int_{t'=t}^{\infty} P_{A}(T_{AC}=t'\cap t'<T_{AI})dt' \\ +\int_{0}^{t} P_{A}(T_{AC}=t'\cap t'<T_{AI})dt'*\int_{0}^{t} P_{V}(T_{VC}=t'\cap t'<T_{VI})dt' \end{matrix}$$

For clarification, each term above is analogous to a cumulative distribution function of RTs. The main difference is that the cumulative distribution function (i.e., *F(t)* or 1- *F(t)* from above), is now weighted by a probability of being correct or incorrect in a certain condition, such as auditory-only trials. In other words, one may simply obtain the distribution of RTs from auditory-only trials, compute the cumulative sum of the distribution (which should sum to 1 when normalized), and then multiplying that cumulative sum by the overall probability correct. (We use ‘*’ to denote a product).

As an example, consider the term: $\int_{0}^{t} P_{A}(T_{AC}=t'\cap t'<T_{AI})dt'$. This is equivalent to the cumulative distribution function from auditory-only trials in which the participant correctly identifies the auditory target before an incorrect alternative, multiplied by the overall probability correct on auditory trials. *P_A_*(‘ ‘) denotes the probability of being correct on auditory-only trials (the probability that the word presented in the auditory domain, *T_AC_*, is recognized before an incorrect word is denoted by *T_AI_*).

Next, consider another term in the equation:$\int_{t'=t}^{\infty} P_{V}(T_{VC}=t'\cap t'<T_{VI})dt'$. This term is equal to the probability that the listener will make a correct V-only identification of the stimulus by a certain time *t* multiplied by the probability correct on visual-only trials (although that a correct response has not been made by time *t*). This term is similar to 1-*F(t)* (the probability that processing has not finished by a certain time), except that it begins at the overall probability correct on visual-only trials instead of 1. *P_V_*(‘ ‘) denotes the probability of being correct on V-only trials (again, the probability that the word actually presented, *T_VC_*, is recognized in the visual domain before an incorrect word, *T_VI_*). Finally, the same is true for *P_AV_*(‘ ‘) which represents the probability of being correct on audiovisual trials. Lastly, the terms *P_A_(I)* and *P_V_(I)* denote the probability of being incorrect on auditory and visual-only trials respectively.

*C_I(t)*, similar to *C(t)*, is a continuous measure computed by obtaining the logarithm of each side of the above equation and dividing by the prediction (in the numerator) by the obtained AV data, $\int_{0}^{t} P_{AV}(T_{AVC}=t'\cap t'<T_{AVI})dt'$in the denominator. Faster and/or more accurate responses on AV trials compared to A and V-only trials yields *C_I(t)* values greater than 1, and indicates “good” audiovisual speech integration.

$$C\_I(t)=\frac{\log\left[\begin{matrix} \int_{0}^{t} P_{A}(T_{AC}=t'\cap t'<T_{AI})dt'*P_{V}(I)+\int_{0}^{t} P_{V}(T_{VC}=t'\cap t'<T_{VI})dt'*P_{A}(I) \\ +\int_{0}^{t} P_{A}(T_{AC}=t'\cap t'<T_{AI})dt'*\int_{t'=t}^{\infty} P_{V}(T_{VC}=t'\cap t'<T_{VI})dt'+\int_{0}^{t} P_{V}(T_{VC}=t'\cap t'<T_{VI})dt'*\int_{t'=t}^{\infty} P_{A}(T_{AC}=t'\cap t'<T_{AI})dt' \\ +\int_{0}^{t} P_{A}(T_{AC}=t'\cap t'<T_{AI})dt'*\int_{0}^{t} P_{V}(T_{VC}=t'\cap t'<T_{VI})dt' \end{matrix}\right]}{\log\left[\int_{0}^{t} P_{AV}(T_{AVC}=t'\cap t'<T_{AVI})dt'\right]}$$

Appendix B

| AV | Take your medicine |
| --- | --- |
| AV | Buy those snowboots |
| AV | Do not scratch your rash or it will just become worse |
| AV | Do not be late today |
| AV | Can the plumber fix the leaking faucet |
| AV | Put both cars in the garage before it starts to snow |
| AV | Alcohol can damage your liver |
| AV | We need to renovate the beach house this summer |
| AV | Computers save time |
| AV | Don’t fool around on the high diving board because it’s dangerous |
| AV | Get out the snow shovel |
| AV | The only way to diet is not to eat |
| AV | Did anyone sing at your wedding reception |
| AV | Do not add too much salt to the soup |
| AV | When you're in London make sure you eat fish and chips |
| AV | Are musicals popular |
| AV | Where did he buy that gray suit |
| AV | Clean the fish tank before you buy those goldfish |
| AV | Pass the ball to him |
| AV | where are all the employee time cards |
| AV | How much is that black dress in the window |
| AV | Buy a new garage door |
| AV | Where's the nurse |
| AV | Does labor day always fall on the first Monday of September |
| AV | Please do not change that radio station |
| A | Have you and your fiancée set a date for the wedding |
| A | Is she wearing the blue dress to the theater |
| A | Clean the cassette player before using it |
| A | When should we have the awards dinner |
| A | Where are the newlyweds going to spend their honeymoon |
| A | Do the ski gloves fit |
| A | He swims fast |
| A | The weather forecast for tomorrow calls for sunshine and low humidity |
| A | Did you warm up the baby’s bottle of milk |
| A | Did the office furniture arrive |
| A | Rents are high |
| A | We used to collect rocks and shells when we were young |
| A | Dancers have a good sense of rhythm |
| A | Isn’t this coffee too sweet |
| A | Polish your shoes |
| A | The bee stung the little girl while she was picking flowers |
| A | The school is closed for Labor day |
| A | The value of the dollar fell with the deficit |
| A | Ask her father |
| A | Quit your job if you are not satisfied with your salary |
| A | Put snow tires on the car today |
| A | If she is not careful shell get a sunburn |
| A | Well pay the telephone bill |
| A | He should floss his teeth |
| A | Has spring arrived |
| V | What will we make for dinner when our neighbors come over |
| V | Is your sister in school |
| V | Does your boss give you a bonus every year |
| V | Don’t spend so much on new clothes |
| V | What’s your recipe for cheesecake |
| V | Is your nephew having a birthday party next week |
| V | What’s the humidity |
| V | Let the children stay up for Halloween |
| V | He plays the bass in a jazz band every Monday night |
| V | How long does it take to roast a turkey |
| V | Which team won |
| V | Take your vitamins every morning after breakfast |
| V | People who invest in stocks and bonds now take some risks |
| V | Those albums are very old |
| V | Aren’t dishwashers convenient |
| V | Is it snowing or raining right now |
| V | The school will be closed for Washington’s Birthday and Lincolns Birthday |
| V | Your check arrived by mail |
| V | Professional musicians must practice at least three hours everyday |
| V | Are whales mammals |
| V | Did the basketball game go into overtime |
| V | When he went to the dentist he had his teeth cleaned |
| V | Well plant roses this spring |
| V | I always mail in my loan payments on time |
| V | Sneakers are comfortable |
